# Using no-cost mobile phone reminders to improve attendance for HIV test results: a pilot study in rural Swaziland

**DOI:** 10.1186/2049-9957-2-12

**Published:** 2013-06-14

**Authors:** Merav Kliner, Abigail Knight, Canaan Mamvura, John Wright, John Walley

**Affiliations:** 1 Nuffield Centre for International Health & Development, University of Leeds, Leeds Institute of Health Sciences, Charles Thackrah Building, University of Leeds, 101 Clarendon Road, Leeds LS2 9LJ, UK; 2 Good Shepherd Hospital, PO Box 2, Siteki, Swaziland; 3 Bradford Institute for Health Research, Bradford Royal Infirmary, Duckworth Lane, Bradford BD9 6RJ, UK

**Keywords:** HIV infections, Mobile phone, Africa, Rural health, Text messaging, Buzzing, MHealth

## Abstract

**Background:**

Mobile technology has great potential to improve adherence and treatment outcomes in healthcare settings. However, text messaging and phone calls are unaffordable in many resource-limited areas. This study investigates the use of a no-cost alternative mobile phone technology using missed calls (‘buzzing’) to act as a patient reminder. The use of missed calls as a patient reminder was evaluated for feasibility and effectiveness as an appointment reminder in the follow-up of newly-diagnosed human immunodeficiency virus (HIV) positive patients in an HIV testing and counselling department in rural Swaziland.

**Methods:**

This pilot study uses a before-and-after operational research study design, with all patients with mobile phones being offered the intervention. The primary outcome was the rate of attendance at the HIV testing and counselling department for collection of results in those with mobile phones before and after the introduction of the intervention.

**Results:**

Over two-thirds, 71.8% (459/639), of patients had a mobile phone. All patients with a mobile phone consented to being buzzed. There was no difference in attendance for follow-up at the clinic before and after the intervention was implemented (80.1% versus 83.3%, p = 0.401), or after adjusting for confounding factors (OR 1.13, p = 0.662).

**Conclusion:**

This pilot study illustrates that mobile technology may be feasible in rural, resource-poor settings as there are high rates of mobile phone ownership and the intervention had a 100% uptake rate, with positive feedback from staff and patients. In this particular setting, the intervention did not improve attendance rates. However, further research is planned to investigate the impact on adherence to appointments and medications in other settings, such as HIV chronic care follow-up and as part of an enhanced package to improve adherence.

## Multilingual abstracts

Please see Additional file 1 for translation for the abstract into the six official working languages of the United Nations.

## Background

Non-attendance at outpatient clinics can lead to poor health outcomes and high costs. There are healthcare- and patient-related factors that can lead to appointments being missed. Healthcare-related factors include poor communication, duration of time between appointments and poor facilities in waiting areas, such as lack of confidentiality [1-3]. Patient-related factors include health beliefs, difficulty with transportation and forgetfulness [4-6]. Any form of reminder may, therefore, reduce the rate of missed appointments.

Mobile technology is frequently used as a tool to improve adherence and treatment outcomes in healthcare settings. It has been used to increase knowledge [7], improve health outcomes [8-11], encourage behaviour change [12] and improve collaboration between healthcare workers in resource-limited settings [13]. A recent Cochrane review considered the impact of text messaging on outpatient attendance [14]. Four randomised control studies were included, all set in high- and middle-income countries: two were set in the UK [15],[16], one in Malaysia [17] and the other in China [18]. The review found that text message reminders improved attendance compared with no reminders (relative risk [RR] = 1.10, 95% confidence interval [CI] 1.03-1.17) and was no better or worse than a phone call. It is likely that factors leading to attendance in clinics will be different in high-income countries as opposed to low- and middle-income countries due to differences in health services provided and general infrastructure. Leong et al. studied mobile phone reminders compared with phone calls in primary care in Malaysia, which found that for every nine text message reminders sent, there was one additional attendance [17]. The text messaging reminder system costs less than half of the mobile phone reminder per attendance. Chen et al. also found similar attendance rates for text messages and phone calls, although text messages were significantly less expensive [18]. None of the studies investigated the reason for non-attendance and all studies stated the importance of high levels of mobile phone ownership.

One randomised control trial (RCT) has been published investigating the impact of mobile phone reminders on outpatient attendance in Kenya; no other RCTs have been identified from low-income countries. Odeny et al. investigated the use of text messaging to improve attendance at post-operative clinic visits after adult male circumcision for HIV prevention [19]. Patients were more likely to return for follow-up after text message reminders than those who did not receive reminders (RR = 1.09, 95% CI 1.00–1.20; p = 0.04). The study also identified that the higher cost of travel to the clinic led to reduced attendance.

Crankshaw et al. and Kunutsor et al. found that mobile phone ownership is between 60-80% in low-income countries, with patients being willing to be contacted verbally or by text message [20],[21]. Therefore, phone reminders may be feasible in these settings. In addition, staff working in resource-poor settings broadly support the use of mobile phone technology [22],[23]. Although there is some evidence of the use of mobile phones to improve health (mobile Health or mHealth) in resource-poor settings, there are few evaluations of the impact of mobile technology on health, with systematic reviewers calling for further evidence on this [10],[14],[24],[25].

These studies suggest that mobile technology may be a useful tool to improve attendance at outpatient service clinics. However, in resource-limited settings, the cost of text messaging may be a barrier to implementation. Text messages in Swaziland, as in the rest of the world, are relatively cheap. In Swaziland, a single text message costs 0.80SZL (£0.06, 1 GBP = 13.99 SZL, 30/4/2013). Although this is a cheap intervention, funding within Swaziland’s health system, as in many developing countries, is limited and often intermittent [26]. Health funding is often found through external donor sources, although these are also not reliable as they are usually associated with larger programmes and may not cover costs of mobile technology. During the course of this study, hospital departments frequently ran out and were not able to replenish ‘airtime’ (mobile phone credit) to conduct follow-up. This outlines the benefit of an intervention that will continue to work regardless whether there is regular funding.

A no-cost alternative to text messaging is using a missed call to act as a reminder. This practice is common in the general population in Africa, South Asia and South America as it is free for both the caller and the receiver. It has different names in different countries, including ‘beeping’ , ‘missed calling’ , ‘flashing’ , ‘pranking’ , ‘lost calling’ and ‘fishing’ [27],[28]. In Swaziland, this practice is called ‘buzzing’ and this is the term that will be used throughout this paper.

Swaziland is facing a severe public health crisis with the highest estimated national prevalence of human immunodeficiency virus (HIV) at 26% among adults aged 15–49, and tuberculosis (TB) incidence rates at 1,287 TB cases/100,000/year, with HIV/TB co-infection adding a particular challenge [29],[30]. This study was based in the regional hospital for the rural region of Lubombo, Swaziland. Diagnosis and management of HIV in the region is conducted by the regional hospital and community-based clinics spread throughout the region. It is coordinated and supported by the Communities Department based at the regional hospital, in conjunction with a number of non-government organisations (NGOs).

Although access to treatment for HIV is improving, there are still considerable challenges in the delivery of services, in particular in relation to follow-up of HIV patients. Within the regional hospital, around 20% of patients who test for HIV fail to collect results and are lost to follow-up. Within the regional hospital, the majority of patients are tested within the HIV testing and counselling department, although some are tested within the maternal and child health clinic, the TB clinic or as an inpatient.

In an attempt to increase the number of patients returning to collect blood results after initial diagnosis with HIV, a mobile phone reminder system using buzzing technology was implemented. A specialised phone which sends automated buzzes to patients the day before appointments was supplied to the HIV testing and counselling department. The aim of this study was to measure the feasibility and effectiveness of buzzing patients as a mobile phone reminder on follow-up attendance to collect blood results (CD4) in the HIV testing and counselling department of a rural hospital in Swaziland.

## Methods

### Study setting

The study was conducted within the HIV counselling and testing department of the Good Shepherd Hospital, a regional hospital in rural Lubombo which serves around 210,000 people. This department was chosen due to the short duration between first and second appointments, which allows the study to investigate the feasibility of using buzzing as a patient reminder.

### Study design

This pilot study uses a before-and-after operational research study design. The intervention was implemented in March 2012, and the attendance rate was compared for the six months before (September 2011 to February 2012) and six months after (March to August 2012) the implementation of the intervention.

### Study population

All patients attending HIV testing and counselling services at Good Shepherd Hospital were included in the study. All patients with a mobile phone were offered the intervention.

### Intervention

After a positive rapid test for HIV, patients have blood tests taken for CD4 count in the HIV testing and counselling department. Patients are asked to return to the department within three to seven days to collect their results. Patients with a very low CD4 count may be followed up by phone call, but most patients who failed to return were previously not routinely followed up. Patients with a mobile phone who consented to participate in the study were all included.

A specialised phone was supplied to the HIV testing and counselling department which automatically buzzes the patient as a reminder the day before their appointment. The system relies on the administrative or counselling staff, when booking the patient for a new appointment, to undertake a number of steps. First, they need to save the number of the clinic’s phone in the patient’s phone as a contact under the name of ‘Go to hospital’ (generally in SiSwati), or as something else agreed upon with the patient to allow patient privacy and confidentiality. The team then inputs the patient’s phone number and appointment date in the specialised clinic phone. The day before the appointment, the patient will be buzzed and the message ‘Go to hospital’ or alternative will be displayed on the patient’s phone.

### Outcome measures

The primary outcome was the rate of attendance at the HIV testing and counselling department for collection of CD4 results in those with mobile phones before and after the introduction of the intervention. The secondary outcomes were: rate of attendance at HIV testing and counselling departments for collection of CD4 results in all patients (those with and without mobiles) attending the department before and after the introduction of the intervention, and time from diagnosis to follow-up appointment.

### Data collection and analysis

Data collection was conducted using the routine registers by hospital staff and anonymised data was entered into an electronic database. The distance travelled to the clinic was calculated based on the patient’s physical address using the TomTom Route Planner and consensus from two local motorcycle adherence officers with a good knowledge of the locality.

Separate analyses were undertaken for those who had mobile phones (per protocol) and all those attending the department before and after, regardless of whether they had a mobile phone or not (intention to treat). The baseline characteristics of those with mobile phones before and after were compared for differences using the Chi-Squared Test and Independent Samples T-Test, as appropriate. The proportion of patients returning to collect CD4 results was compared in the before and after groups, including confidence intervals (CI) around the rates and a Chi-Squared Test for independence. Multiple logistic regression was conducted for the primary outcome to adjust for confounding. The mean time to collect CD4, as well as CI around the mean, were calculated. Analysis was undertaken using SPSS Statistics Version 19 software and Microsoft Excel.

### Sample size

Three hundred patients with mobile phones were seen in the HIV testing and counselling services department in the six months before the intervention was implemented, with a re-attendance rate of 80%. Using alpha of 0.05, power of 0.8 and assuming the same number of patients attending in the six months after the intervention, the study is powered to detect a 9% difference, increasing attendance from 80% to 89%.

### Ethics

Ethics approval for this study was granted by the Swaziland Ministry of Health Ethics Committee.

## Results

Over 400 (417) patients tested positive for HIV in the six months before the intervention and 222 tested positive in the six months after the intervention in the HIV testing and counselling department. Over two-thirds, 71.8% (459/639), of patients had a mobile phone – 71.2% in the before group and 73.0% in the after group. The baseline characteristics of patients with mobile phones in the before and after groups are summarised in Table 1.

**Table 1:** Baseline characteristics of patients with mobile phones in the before and after groups

	**Before**	**After**	**P value**
**Female,% (n)**	54.2% (161)	54.3% (88)	0.98
**Age in years, mean (standard deviation)**	31.8 (13.0)	33.1 (10.9)	0.27
**Distance from home to hospital, mean (standard deviation)**	37.7 km (33.3)	29.4 km (28.2)	0.008
**Eligible for ART,% (n)**	58.6% (174)	60.5% (98)	0.69

All patients with a mobile phone consented to being buzzed. Of those with mobile phones, 83.3% (135/162, 95% CI 77.6-89.1%) of patients returned to the HIV testing and counselling department to collect CD4 results in the six months after the implementation of the intervention, compared with 80.1% (238/297, 95% CI 75.6-84.7%) in the six months before the intervention (p = 0.401) (see Table 2). After adjustment for baseline characteristics, the adjusted OR was calculated as 1.13, although this was not statistically significant (p = 0.662). The mean number of days taken to collect CD4 results using per protocol analysis was four days in the before group and six days in the after group (p = 0.002).

**Table 2:** Per protocol and intention to treat results of attendance before and after intervention

	**Attendance in 6 months before intervention,% (95% CI)**	**Attendance in 6 months after intervention,% (95% CI)**	**P value**
**Per protocol**	80.1% (75.6%-84.7%)	83.3% (77.6%-89.1%)	0.401
**Intention to treat**	83.0% (79.4%-86.6%)	82.4% (77.4%-87.4%)	0.863

When analysed using an intention to treat analysis, including all cases with or without mobile phones, 82.97% (346/417) collected CD4 results before the intervention, and 82.43% (183/222) collected the results after the intervention, was implemented (p = 0.863).

Patients who collected CD4 results tended to be older, male, live closer to the hospital and be eligible for initiation of antiretroviral therapy (ART). Differences between patients collecting CD4 results and those not collecting results are presented in Table 3.

**Table 3:** Characteristics of patients collecting CD4 and not collecting CD4

	**Collecting CD4**	**Not collecting CD4**
Age (mean)	36	34
Male sex (%)	46.5%	44.5%
Distance to GSH, km (mean)	33	37
Eligible for ART (%)	65.8%	47.3%

## Discussion

We found no significant difference in the rate of attendance of patients for their CD4 results after the introduction of no-cost mobile phone reminders. One hypothesis was that forgetfulness was the main reason for non-attendance, which is in line with the literature on the use of mobile phone reminders for appointments [6],[31-36], and therefore phone reminders would help with this. However, if the assumption is that forgetfulness is a key factor in non-attendance, the short timeframe in which patients are expected to come back for appointments may have limited the measurable benefit of a reminder system. The average number of days between the two appointments is four to six days and, therefore, the reason for non-attendance may not be failing to remember to attend.

Patients who collected CD4 results tended to be older, male, live closer to the hospital and be eligible for initiation of ART. Younger age has previously been suggested as a risk factor for non-attendance [37]. Generally, male sex is associated with poorer attendance [37] and, therefore, the finding in this study may be related to cultural beliefs. Patients who live further from the hospital have been found not to attend their appointments, in line with existing literature [4],[19],[33]. This is particularly likely to impact patients in rural Swaziland as some patients were required to travel distances over 100 km to reach the clinic. Patients who were eligible for initiation of ART were also more likely to attend their appointments. This may be because patients were symptomatic and therefore concerned to find out their results. Additional studies may be required to further understand reasons for non-attendance in this setting as information on patients’ reasons for non-attendance was not collected during this study and is thus a limitation of the study. A further limitation of the study is that it does not investigate patients’ prior knowledge, experience and expectations of healthcare, and HIV counselling and testing in particular, which would be helpful to understand reasons for non-attendance.

As was reported in the cited literature from resource-limited settings [20],[21], mobile phone ownership is high in Swaziland, with over 70% of patients owning a mobile phone. In addition, all patients who owned a mobile phone consented to enter the study and receive the reminder buzz. This shows that being sent a reminder message was acceptable to all patients. A large number of returning patients reported to staff at the HIV testing and counselling department that they were grateful for the reminder. Staff at the HIV testing and counselling service found the technology easy to use and reported that they would be happy to continue using the service at the completion of the study. This suggests that the use of buzzing is feasible and acceptable in this setting.

There were a number of differences in the before and after groups, which are largely due to the operational nature of this research being conducted in a real-world setting against a background of a rapidly changing national emergency. The number of patients testing positive before the implementation of the intervention was higher than after the intervention was in place. This is likely to be due to changes in the way services were being delivered at the hospital, with an emphasis on decentralisation of HIV testing and counselling, and integrating testing into general services such as outpatients, the TB department, and the maternal and child health department. During the course of this study, a new HIV counsellor was recruited to work within the outpatient department and this is likely to have decreased the number of patients attending the stand-alone HIV testing and counselling department. There were more patients with unknown addresses after the intervention compared with before, 16% (26) versus 1% (3), preventing the calculation of their distance from hospital in these patients. This was due to the adoption of a new data management system for HIV services in the hospital soon after the implementation of the intervention. There were a number of concerns about data completeness soon after the adoption of the data system, which were rectified quickly but led to gaps in data collection.

The mean number of days taken to collect CD4 was higher in the group that received the intervention compared with the before group. There is no clear explanation of why this may be, but it may be due to chance as the study is not powered to detect a significant difference in the secondary outcome. As this is a before-and-after study, this difference may also be due to external factors such as the changes in the economic climate, which has been deteriorating within Swaziland and has led to public sector and transport strikes perhaps providing a barrier to access and leading to difficulties paying for travel.

Almost a third (30%) of patients accessing HIV testing did not have a mobile phone. Differences between those with a mobile phone and those without are presented in Table 4. Those without a mobile phone are slightly older, which is consistent with social findings that younger people take up technology faster than the older generation [38]. Fewer men than women have mobile phones, which goes against the general understanding that men would have faster uptakes of technology than women [38]. People with mobile phones tended to live, on average, further from the hospital. The region is very rural and it is difficult to make any assertions from this. Lastly, higher proportions of people without mobiles require initiation of ART. This may relate to deprivation as people that cannot afford mobile phones may be less likely to travel to health services and seek health care.

**Table 4:** Characteristics of patients with and without mobile phone

	**With mobile**	**Without mobile**
Age (mean)	32	36
Male sex (%)	45.4%	50.3%
Distance to GSH (mean)	35	28
Eligible for ART (%)	60.2%	70.1%
Total	482	157

The sample size required to power the study to detect a 9% difference was not reached due to the limited numbers of newly-diagnosed HIV positive individuals. With 297 patients in the before group, 162 in the after group, an alpha of 0.05 and a power of 0.8, the study was powered to detect a 10% increase in attendance from 80% to 90%, not quite the 9% difference that was aimed for.

### The role of a no-cost messaging system in a low-resource setting

To our knowledge, this is the first study considering the use of buzzing in a health setting as a patient reminder. This study illustrates that, in line with current literature, mobile phone ownership is high in rural Swaziland, and patients and staff were happy to receive reminders by mobile phone. Automated buzzing as a mobile phone reminder is a relatively new technology and has been shown, in this study, to be popular with patients and staff as it is in line with routine daily practice for society. Although this study did not show a benefit in this setting, feedback illustrates that this technology is likely to be acceptable to the patient population and healthcare workers of Swaziland.

Text messages in Swaziland are cheap. However, the HIV testing and counselling department sees over 250 patients per month. Assuming 70% of those have a mobile phone, 175 reminders would be required to be sent a month from this department, amounting to E140 (£10.50) a month. Although this is not a significant sum, as funding is not consistent, phone calls or text messages for follow-up is not a reliable mode of communication. This was illustrated during the course of this study, where hospital departments frequently did not have ‘airtime’ (mobile phone credit) to conduct follow-up. This outlines the benefit of an intervention that will continue to work regardless whether there is regular funding. There are, however, additional costs to consider including the cost of the phone, software development and staff time. The software has been developed and phone donated by an American philanthropist and, therefore, no direct costs were associated with this intervention. The software development team are in the process of developing a mobile phone application (app), which will be freely downloadable and can be used by any healthcare provider worldwide. Increasingly, mobile phones used by people in Africa are able to access the internet [39], and this allows for the new technology to be downloaded onto a suitable smart phone and used in any setting. Therefore, this will limit costs for the healthcare setting. Staff did not feel that they spent additional time on this new intervention compared to follow-up using text messages or phone call reminders and, therefore, this is not perceived to be a barrier to implementation.

While we did not demonstrate benefits of improved attendance for test results using buzzing as a short-term reminder, it is possible that the technology could have other applications. Buzzing could be used as a prompt for adherence to medications e.g. daily reminder to take antiretroviral or TB medications; in chronic disease clinics, such as HIV antiretroviral clinics or non-communicable disease (NCD) clinics; and where time between appointments is between one and three months and therefore, forgetting appointments is likely to be a more common reason for missed appointments. A larger study to evaluate the effect of buzzing in chronic HIV care is being planned in this setting. In addition, adherence strategies in HIV care often have relatively small effects, although may additively provide benefits [40]. Therefore buzzing is being planned into a larger study of developing a package of interventions to improve adherence and treatment outcomes in NCD control and multi-drug resistance TB in this setting.

## Conclusion

This study failed to detect any difference in attendance after providing mobile phone reminders using buzzing. However, it does suggest that using mobile phone technology in rural Swaziland may be feasible and acceptable. The main barrier to using mobile phone technology can be cost and, therefore, using a free service such as buzzing may be an affordable alternative to phone calls or text messaging. Further study in chronic care settings and as part of adherence packages is planned to investigate if buzzing can improve attendance and adherence in resource-limited settings.

## Abbreviations

HIV: Human immunodeficiency virus; TB: Tuberculosis.

## Competing interests

The authors wish to declare that they have no competing interests.

## Authors’ contributions

MK was involved in the conception, design, acquisition and analysis of data, and of the drafting the manuscript. AK was involved in the acquisition of data and critically revised the manuscript. CM was involved in the design of the study and critically revised the manuscript. JWr was involved in the conception and design of the study and critically revised the manuscript. JWa was involved in the design of the study and critically revised the manuscript. All authors read and approved the final manuscript.

## Supplementary Material

Additional file 1 Translation of the abstract into the six official working languages of the United Nations. Click here for file
