# Supplementary material for: Using no-cost mobile phone reminders to improve attendance for HIV test results: a pilot study in rural Swaziland
Source: Infect Dis Poverty. 2013 Jun 14;2:12. doi: 10.1186/2049-9957-2-12 (PMC3710074; doi:10.1186/2049-9957-2-12)
Supplement: Additional file 1 — Translation of the abstract into the six official working languages of the United Nations. [file 2049-9957-2-12-S1.pdf]

Translation of the abstract into the six official working languages of the United Nations

استخدام رسائل التذكير بالهواتف المحمولة لتحسين نسبة الحضور للحصول على نتائج اختبارات "فيروس نقص المناعة HIV": دراسة تجريبية بالمناطق الريفية بسوازيلاند

ميراف كلينر، أبيجيل نايت، كنعان مامفورا، جون رايت، جون والى

ملخص

معلومات عامة: لتقنيات الهاتف المحمول إمكانية لتحسين نتائج الإلتزام والعلاج في أطر الرعاية الصحية. إلا أن الاستعانة بالرسائل النصية والمكالمات الهاتفية، لا يمكن تحمل تكلفتها بالمناطق محدودة الموارد. تبحث هذه الدراسة استخدام بديل بدون تكلفة لتقنية الهواتف المحمولة، وهو استخدام المكالمات الفائتة ("الرنين") كمذكر للمرضى. أثبت تقييم الاستعانة بالمكالمات الفائتة كمذكر للمرضى، جدوى وفعالية كمذكر بالمواعيد لمتابعة المرضى الذين تم تشخيصهم حديثاً كحاملين لفيروس نقص المناعة، وذلك بقسم الاختبارات والاستشارات بالمناطق الريفية بسوازيلاند.

المناهج: تستخدم هذه الدراسة تصميم الدراسة البحثية لما قبل وبعد التنفيذ، مع جميع المرضى ممن لديهم هواتف محمولة والذين قُدمت لهم هذه الوسيلة. وأظهرت النتائج الأولية نسبة الحضور بقسم الاختبارات والاستشارات للمرضى الذين حضروا من أجل الحصول على نتائج الاختبارات ممن لديهم هواتف محمولة، وذلك قبل وبعد استخدام هذه الوسيلة.

النتائج: لدى أكثر من ثلثي المرضى 71.8% (459/639) هواتف محمولة. وافق جميع المرضى ممن لديهم هواتف محمولة على استخدام وسيلة المكالمات الفائتة. لم يكن هناك اختلاف بين نسبة الحضور للمتابعة بالعيادة قبل وبعد استخدام هذه الوسيلة (80.1% مقابل 83.3%، p=0.401)، أو بعد التعديل نظراً لعوامل خارجية (أو 1.13، p=0.662).

الاستنتاج: تظهر هذه الدراسة التجريبية أن تقنية الهواتف المحمولة يمكن أن تكون ذات جدوى في إطار المناطق الريفية فقيرة الموارد، نظراً لارتفاع عدد مالكي الهواتف المحمولة، ووصول نسبة القبول لاستخدام هذه الوسيلة إلى 100%، مع تقديم الموظفين والمرضى لمعلومات مرتدة إيجابية. في هذه الأطر الخاصة، لم يعمل استخدام هذه الوسيلة على تحسين نسبة الحضور. ومع هذا فهناك تخطيط لإجراء بحث إضافي لتقصي تأثير الإلتزام بالمواعيد والأدوية في أطر أخرى، مثل متابعة الرعاية المستديمة لفيروس نقص المناعة، وكجزء من مجموعة مطورة للتحسين من الإلتزام.

Translated from English version into Arabic by Raneya Hassan, through

TRANSLATORS WITHOUT BORDERS

利用无成本手机提醒改善 HIV 检测的参与：在斯威士兰农村的试点研究

Merav Kliner, Abigail Knight, Canaan Mamvura, John Wright, John Walley

摘要

引言：移动技术在提高依从性和医疗设备的治疗效果方面有很大的潜力。然而，在很多资源有限的地区，人们连短信和电话都负担不起。本研究探讨利用无成本的替代手机技术将未接来电（"嗡嗡"声）作为对患者的一个提醒。在斯威士兰农村，评估了使用未接来电作为对新诊断为 HIV 阳性患者的定期复查和去咨询部门的提醒的可行性和有效性。

方法：本试点研究使用了前后对比研究设计，对所有拥有手机患者提供干预。主要结果为在使用手机干预前后患者在 HIV 检测和咨询部门的参与率。

结果：超过 2/3 的患者（71.8%，459/639）拥有一部手机。所有拥有手机的患者同意接受未接来电。在实施干预措施前后（80.1% versus 83.3%, p=0.401），或在调整干扰因素后（OR 1.13, p=0.662），门诊定期复查的参与情况无显著差异。

结论：本试点研究显示移动技术在农村和资源匮乏的地区可能是可行的，因为那里的手机拥有率很高，且干预措施的接受率达 100%，工作人员和患者反馈积极。在这种特殊的环境中，干预并未提高参与率。然而，计划对其他环境下坚持使用约定和用药的影响进行深入研究，如 HIV 慢性护理随访和作为加强包的一部分来提高依从性。

Translated from English version into Chinese by Yang Pin, through

CHINESE CENTER FOR DISEASE CONTROL AND PREVENTION
NATIONAL INSTITUTE FOR PARASITIC DISEASES

Utiliser des rappels par téléphone mobile sans frais afin d'améliorer la fréquence des résultats de test de dépistage du VIH : une étude pilote au Swaziland rural

Merav Kliner, Abigail Knight, Canaan Martins, John Wright, John Walley

Résumé

Contexte: La technologie mobile a un grand potentiel pour améliorer l'observance du traitement et ses résultats dans les établissements de soins. Toutefois, la messagerie texte et les appels téléphoniques sont hors prix dans de nombreuses zones à ressources limitées. Cette étude examine l'utilisation d'une autre technologie de téléphonie mobile sans frais en utilisant la « vibration » des appels ratés en guise de rappel au patient. L'utilisation des appels ratés en guise de rappels au patient a été évaluée pour sa faisabilité et son efficacité comme un rappel de rendez-vous dans le cadre d'un suivi de patients séropositifs récemment diagnostiqués avec un virus de l'immunodéficience humain (VIH) à un service de conseils et de dépistage du VIH en Swaziland rural.

Méthodes: Cette étude pilote utilise un concept d'étude avant-après la recherche opérationnelle, qui offre l'intervention à tous les patients ayant des téléphones portables. Le résultat principal était le taux de fréquentation au service de conseils et de dépistage du VIH pour la collecte des résultats chez ceux utilisant des téléphones mobiles avant et après l'introduction de l'intervention.

Résultats: Plus des deux tiers, soit 71,8 % (459/639), des patients possédaient un téléphone mobile. Tous les patients utilisant un téléphone portable ont consenti à recevoir la vibration de rappel. Aucune différence n'a été notée dans la fréquentation pour le suivi à la clinique avant et après la mise en œuvre de l'intervention (soit 80,1 % contre 83,3 %, p = 0.401), ou après l'ajustement en raison des facteurs de confusion (OR 1.13, p = 0,662).

Conclusion: Cette étude pilote montre que la technologie mobile peut être réalisable dans des environnements pauvres en ressources rurales car il y a des taux élevés de personnes en possession de téléphone mobile et l'intervention avait un taux de participation de 100 %, avec des résultats positifs chez le personnel et les patients. Dans ce contexte particulier, l'intervention n'a pas amélioré les taux de fréquentation. Cependant, de plus amples recherches sont prévues en vue d'étudier l'impact sur le respect des rendez-vous et la prise des

médicaments dans d'autres contextes, tels que le suivi de soins chroniques du VIH et dans le cadre d'un module renforcé pour améliorer l'observance.

Translated from English version into French by MultiPro, through

TRANSLATORS
WITHOUT BORDERS

Использование бесплатных напоминаний по сотовой связи для улучшения явки пациентов для выяснения результатов анализов на ВИЧ: пилотное исследование в сельской местности Свазиленда

Мерав Клайнер, Абигейл Найт, Кенаан Мамвура, Джон Райт, Джон Уолли (Merav Kliner, Abigail Knight, Canaan Mamvura, John Wright, John Walley)

Аннотация

Постановка проблемы: Телефонная сотовая связь позволяет значительно улучшить соблюдение медицинских предписаний и повысить эффективность лечения пациентов. Однако во многих слаборазвитых районах передача СМС-сообщений и телефонные звонки слишком дороги. Настоящее исследование посвящено анализу использования пропущенных звонков как бесплатной альтернативы СМС-сообщениям для напоминания. Использование пропущенных звонков для напоминания было проанализировано с точки зрения применимости и эффективности для напоминания о визите к врачу при наблюдении вновь диагностированных ВИЧ-инфицированных пациентов в отделении по тестированию на ВИЧ (вирус иммунодефицита человека) и консультациям в сельской местности Свазиленда.

Пути решения проблемы: В основу методики данного пилотного исследования была положена структура «до и после». Участвовать в эксперименте было предложено всем пациентам с сотовыми телефонами. За первичный результат был принят процент владельцев сотовых телефонов, явившихся в отделение по тестированию на ВИЧ и консультациям для получения результатов до и после начала эксперимента.

Результаты: Примерно две трети пациентов, 71.8% (459/639), имели сотовые телефоны. Все пациенты с сотовыми телефонами согласились получать звонки. Различий в явке в клинику для наблюдения до и после начала эксперимента не было (80.1% против 83.3%, $p=0.401$), или с учетом вмешивающихся факторов (OR 1.13, $p=0.662$).

Выводы: Данное пилотное исследование показывает, что сотовая связь может быть применена в слаборазвитых сельских районах, поскольку процент владельцев сотовых телефонов высок. Эксперимент проходил при стопроцентном участии и с положительными отзывами от медицинского персонала и пациентов. В данном

конкретном случае посещаемость не увеличилась с началом эксперимента. Однако намечено дальнейшее исследование влияния звонков на регулярность визитов к врачу и точное соблюдение медицинских предписаний в других ситуациях, таких, как наблюдение за хроническими ВИЧ-инфицированными больными, а также в качестве составляющей комплексной программы по улучшению явки пациентов.

Translated from English version into Russian by Barking Knitter, through

TRANSLATORS
WITHOUT BORDERS

Las alertas gratuitas por celular como herramienta para mejorar la asistencia a las citas para la entrega de los resultados de las pruebas de detección del VIH: un estudio piloto en las zonas rurales de Suazilandia

Merav Kliner, Abigail Knight, Canaan Mamvura, John Wright, John Walley

Resumen

Antecedentes: En el campo de la salud la tecnología móvil tiene un gran potencial para ayudar a que los pacientes cumplan con los tratamientos y obtengan mejores resultados. Sin embargo, los mensajes de texto y las llamadas pueden ser muy costosos para los pacientes en zonas de escasos recursos. Este studio investiga el uso de una alternativa gratuita: las llamadas perdidas (timbres) como medio para recordarle al paciente que tiene que cumplir una cita. El uso de llamadas perdidas como recordatorio fue evaluado en cuanto a su factibilidad y efectividad en el seguimiento de los pacientes recientemente diagnosticados positivos con el virus de la inmunodeficiencia humana VIH en un departamento de orientación y pruebas médicas en pruebas ubicado en una zona rural de Suazilandia.

Método: Este estudio piloto utiliza un diseño de investigación operacional del tipo "antes y después" y le fue propuesto a todos los pacientes que tuvieran un teléfono celular. El objetivo principal era obtener los porcentajes de asistencia a las citas para reclamar los resultados de los exámenes para detectar el VIH, antes y después de poner en práctica el uso de las llamadas perdidas.

Resultados: Más de dos terceras partes de los pacientes, el 71.8% o 459 pacientes entre 639, tenían teléfono celular. Todos los pacientes con teléfono celular estuvieron de acuerdo con que les timbraran. No hubo diferencia en la asistencia a las citas de seguimiento antes y después de realizada la intervención (80.1% versus 83.3%, $p=0.401$), o después de realizar los ajustes por factores de confusión (OR 1.13, $p=0.662$).

Conclusión: Este estudio piloto demuestra que el uso de la tecnología móvil es posible en zonas rurales de escasos recursos, pues un alto porcentaje de las personas poseen un teléfono celular y la intervención propuesta fue aceptada por el 100% de éstas, con comentarios positivos de los pacientes y de los empleados. En este caso en particular, la intervención no mejoró el promedio de asistencia. Sin embargo, se han proyectado más investigaciones para descubrir el impacto en la asistencia a las citas y el cumplimiento del tratamiento en áreas relacionadas, como el seguimiento a la atención crónica del VIH y como parte de un paquete ampliado para mejorar la asistencia.

Translated from English version into Spanish by Andrés García, through

TRANSLATORS
WITHOUT BORDERS
